# A Rapid-Learning Health System to Support Implementation of Early Intervention Services for Psychosis in Quebec, Canada: Protocol

**DOI:** 10.2196/37346

**Published:** 2022-07-19

**Authors:** Manuela Ferrari, Srividya Iyer, Annie LeBlanc, Marc-André Roy, Amal Abdel-Baki

**Affiliations:** 1 Douglas Research Centre Montreal, QC Canada; 2 Department of Psychiatry McGill University Montreal, QC Canada; 3 Department of Family Medicine Laval University Quebec, QC Canada; 4 Vitam – Centre de recherche en santé durable Laval University Quebec, QC Canada; 5 Department of Psychiatry and Neurosciences Laval University Quebec, QC Canada; 6 Cervo Brain Research Centre Quebec, QC Canada; 7 Centre de Recherche du Centre Hospitalier de l'Université de Montreal (CHUM) Montreal, QC Canada; 8 Department of Psychiatry Université de Montréal Montreal, QC Canada

**Keywords:** rapid-learning health system, early intervention for psychosis, measurement-based care, real-time electronic data capturing, patient-oriented research, knowledge translation, mobile phone

## Abstract

**Background:**

Given the strong evidence of their effectiveness, early intervention services (EIS) for psychosis are being widely implemented. However, heterogeneity in the implementation of essential components remains an ongoing challenge. Rapid-learning health systems (RLHSs) that embed data collection in clinical settings for real-time learning and continuous quality improvement can address this challenge. Therefore, we implemented an RLHS in 11 EIS in Quebec, Canada.

**Objective:**

This project aims to determine the feasibility and acceptability of implementing an RLHS in EIS and assess its impact on compliance with standards for essential EIS components.

**Methods:**

Funding for this project was secured in July 2019, and ethics approval was received in December 2019. The implementation of this RLHS involves 6 iterative phases: external and internal scan, design, implementation, evaluation, adjustment, and dissemination. Multiple stakeholder groups (service users, families, clinicians, researchers, decision makers, and provincial EIS associations) are involved in all phases. Meaningful EIS quality indicators (eg, satisfaction and timeliness of response to referrals) were selected based on a literature review, provincial guidelines, and stakeholder consensus on prioritization of indicators. A digital infrastructure was designed and deployed comprising a user-friendly interface for routinely collecting data from programs; a digital terminal and mobile app to collect feedback from service users and families regarding care received, health, and quality of life; and data analytic, visualization, and reporting functionalities to provide participating programs with real-time feedback on their ongoing performance in relation to standards and to other programs, including tailored recommendations. Our community of practice conducts activities, leveraging insights from data to build program capacity while continuously aligning their practices with standards and best practices. Guided by the RE-AIM (Reach, Effectiveness, Adoption, Implementation, Maintenance) framework, we are collecting quantitative and qualitative data on the reach, effectiveness, adoption, implementation, and maintenance of our RLHS for evaluating its impacts.

**Results:**

Phase 1 (identifying RLHS indicators for EIS based on a literature synthesis, a survey, and consensus meetings with all stakeholder groups) and phase 2 (developing and implementing the RLHS digital infrastructure) are completed (September 2019 to May 2020). Phases 3 to 5 have been ongoing (June 2020 to June 2022). Continuous data collection through the RLHS data capture platforms and real-time feedback to all stakeholders are deployed. Phase 6 will be implemented in 2022 to assess the impact of the RLHS using the Reach, Effectiveness, Adoption, Implementation, and Maintenance framework with quantitative and qualitative data.

**Conclusions:**

This project will yield valuable insights into the implementation of RLHS in EIS, offering preliminary evidence of its acceptability, feasibility, and impacts on program-level outcomes. The findings will refine our RLHS further and advance approaches that use data, stakeholder voices, and collaborative learning to improve outcomes and quality in services for psychosis.

**International Registered Report Identifier (IRRID):**

DERR1-10.2196/37346

## Introduction

Psychotic disorders, which include schizophrenia-spectrum and affective psychoses (bipolar and major depressive disorders with psychosis), have a lifetime prevalence of 3% to 3.5% [1,2] and typically emerge during a major neuro-sociodevelopmental period (age 15-30 years), posing further challenges in the early stages of illness management. Early intervention services (EIS) are now widely recognized as more effective than routine care for the treatment of psychosis [3-5] in the critical first 2- to 5-year period [6]. EIS aim to reduce the duration of untreated psychosis (ie, the delay between the first psychotic symptoms and initiation of adequate treatment), which negatively affects clinical and functional outcomes [7-10], and to positively affect longer-term outcome trajectories by maximizing symptomatic, functional, and recovery outcomes in this critical period. The EIS model was designed to address ubiquitous challenges in treating psychotic disorders, such as poor service engagement, medication nonadherence, and comorbid substance use, which are particularly salient in the early years [6,11]. This period is also associated with maximum risk of tragic outcomes such as violence, social and vocational impairment, long-term disability, and suicide [6,8,12-15].

Many countries [16,17], including Canada, have implemented the EIS model. On the basis of international and national guidelines for quality care, the model includes, among other essential components, an open referral process, timely access to treatment (reduced treatment delay), active engagement of service users and family members encouraged by a youth-friendly atmosphere, and comprehensive team-based care that combines pharmacological treatment using the lowest effective doses of antipsychotic medications with the provision of integrated, evidence-based psychosocial interventions [16,18,19]. Appropriate patient-staff ratios and continuous professional development are also recommended by the model [16,18,19].

In Canada, Ontario and British Columbia have taken the lead in developing EIS policies and creating provincial EIS networks [16,17]. In the late 1990s, clinicians supported by their institutions led the initial development of EIS in Quebec, where this research team is based. This was followed by the creation of the *Association québécoise des programmes pour premiers épisodes psychotiques* (AQPPEP), the Quebec association of EIS, in 2004. Support for the implementation of EIS across jurisdictions is enhanced by continuous professional development; networking; mentoring; communities of practice; and the promotion of evidence-based practices, use of clinical guidelines, and innovation. However, despite these efforts, EIS implementation in Canada [20-22] and internationally [23-25] has long been impeded by a lack of standards in some jurisdictions and implementation challenges related to delivering complex models of care in real-life settings [21,22,26]. Research has identified major challenges in relation to integrating essential organizational components (eg, open referral processes and appropriate patient-case manager ratios) [22] and insufficient funding and mentoring to ensure consistent implementation [22-25], as well as lack of systematic monitoring related to quality-of-care indicators and outcomes [21,22,26].

In 2017, the Quebec Ministry of Health and Social Services invested an additional CAD $10 million (US $7,905,200) to improve existing EIS and develop new services in underserved regions, adding approximately 16 new teams for a total of 33 EIS teams by 2020, which doubled EIS coverage across the province in <3 years. The Ministry of Health and Social Services also published the 2017 *Cadre de référence - Programmes d’interventions pour premiers épisodes psychotiques*, the Quebec guidelines for EIS, providing guidance on the essential components and related indicators for EIS. Although service improvements have been observed since the promulgation of this policy and related funding commitments, gaps remain in the implementation and real-time monitoring of practices related to EIS standards in Quebec [21]. Indeed, a survey conducted with 28 of the 33 Quebec EIS in 2020 revealed that administrative and organizational components such as clinical and administrative data collection, adherence to recommended patient to case manager ratios, and quality assurance monitoring were less widely implemented [21]. Moreover, many EIS were not able to offer some recommended specialized treatments such as cognitive behavioral therapy or peer support, often because of the lack of appropriately trained professionals.

In other fields of medicine, rapid-learning health systems (RLHSs) that embed data collection in clinical settings for real-time learning and continuous quality improvement have been deployed to improve service quality. We designed and piloted an RLHS to support Quebec EIS by systematically collecting real-time data for use in improving service quality and clinical practice.

## Methods

### Objectives

**Figure 1:**
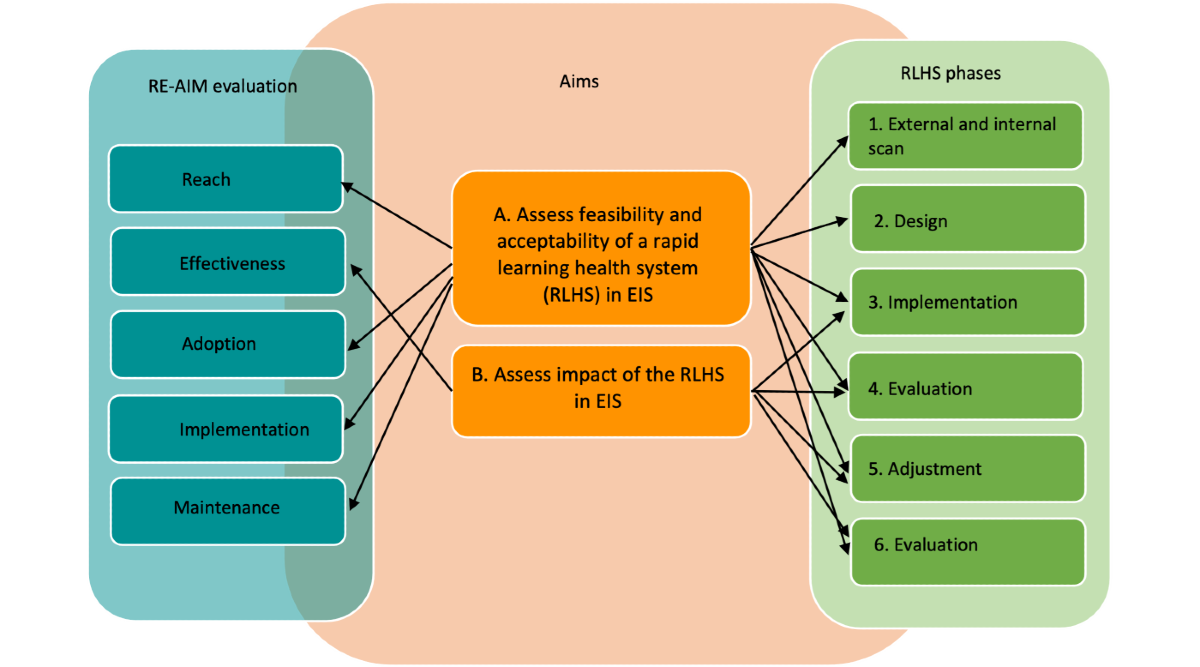
Project conceptual framework. EIS: early intervention services; RE-AIM: Reach, Effectiveness, Adoption, Implementation, and Maintenance framework; RLHS: rapid-learning health system.

The primary objective of this multiphase, mixed methods project is to determine the feasibility and acceptability of implementing an RLHS in EIS. The secondary objective is to evaluate the 2-year impact of the RLHS on patient-, family-, EIS-, and provincial-level outcomes (Figure 1).

More specifically, feasibility and acceptability were evaluated in terms of 2 objectives using the Reach, Effectiveness, Adoption, Implementation, and Maintenance (RE-AIM) framework. Objective 1 investigates the reach, adoption, implementation, and maintenance of (1) a user-friendly electronic platform that captures continuous data on selected service quality indicators from individual EIS, (2) continuous data-informed feedback to EIS, and (3) data-informed and capacity-building activities tailored to EIS members of our RLHS and the overall Quebec EIS community for improving service quality where EIS components are weaker. Objective 2 addresses *effectiveness* by evaluating improvements in (1) adherence to EIS components among participating EIS, (2) capacity of the EIS to collect data for monitoring quality of care, (3) key patient and family outcomes, and (4) program-level and provincial decision-making related to meeting quality-of-care standards in EIS.

### RLHS: A Novel Paradigm in EIS Implementation

The new RLHS health care paradigm [27] has been shown to promote innovation and responsiveness by bridging the gap between evidence and practice and improving efficiency, effectiveness, and quality in health care delivery, primarily in medical health care settings [28-32]. Among the various definitions of RLHS [28-31], the most frequently cited is the Institute of Medicine definition, which envisions “the development of a continuously learning health system in which science, informatics, incentives, and culture are aligned for continuous improvement and innovation, with best practices seamlessly embedded in the delivery process and new knowledge captured as an integral by-product of the delivery experience” [33]. According to the Institute of Medicine, an RLHS uses digital technologies to (1) generate and apply the best evidence to support collaborative health care choices by patients and providers; (2) drive the discovery process as a natural outgrowth of patient care; and (3) ensure quality, safety, value, and innovation in health care [33,34]. Digital technology, hardware, and software that process and transmit digital information (eg, electronic health records, databases, analytic tools, and visual dashboards) are at the core of the RLHS, providing data and information as catalysts for system *learning* and the transformation of clinical practice.

The RLHS addresses the knowledge-to-practice gap in medical care through the rapid and ethical transfer of knowledge produced by clinical research into routine clinical practice [35,36]. The RLHS can foster a culture of shared responsibility between clinicians and patients [37,38] and facilitate engagement by patients, clinical teams, and program managers for the production and dissemination of evidence to the public [39]. Thus, an RLHS was chosen as an innovative research paradigm to guide the transformation of the Quebec EIS system by addressing previously identified gaps such as lack of or inconsistent monitoring of quality and performance and gaps between standards, evidence, and actual practice.

This study, conducted in partnership with EIS and key stakeholders, is grounded in principles of patient-oriented research that support meaningful and active engagement by patients and families. Adhering to this framework, we invited participation by patients, families, and knowledge users (eg, program administrators, clinicians, and representatives of the *Centre national d’excellence en santé mentale* of the Quebec Ministry of Health and Social Services mental health advisory branch) to develop the study (eg, study design and choice of outcomes), and we will continue this practice in the implementation and dissemination of the study findings.

**Figure 2:**
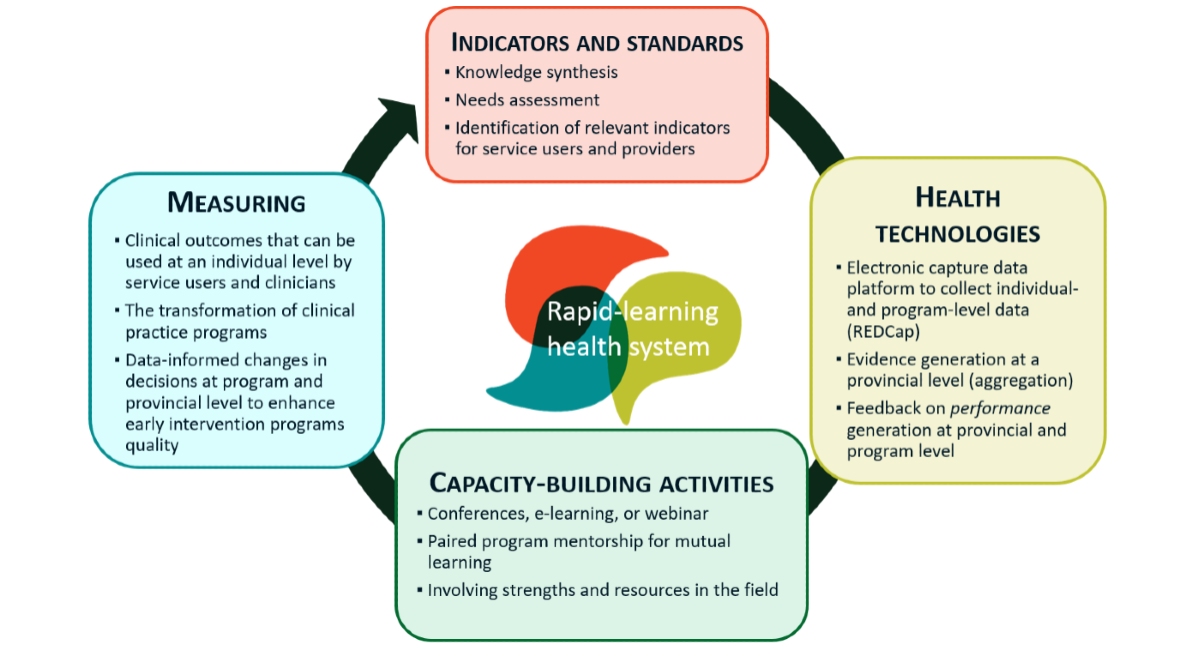
Rapid-learning health system for early intervention for psychosis.

Guided by the literature [27,33,34], the implementation of our RLHS involves 6 iterative phases (objective 1), as shown in Figure 2. These phases are outlined in Textbox 1.

Guided by the RE-AIM framework, we are in the process of collecting quantitative and qualitative data on the reach, effectiveness, adoption, implementation, and maintenance of the RLHS. These RE-AIM data will be analyzed to evaluate the impact of the RLHS and address the 2 study objectives.

**The 6 iterative phases of implementation in our rapid-learning health system (RLHS).**

**Implementation phases**

1. Identification of indicators in the RLHS for early intervention services (EIS) through an external and internal scan and building of the RLHS community. This involves a knowledge synthesis of relevant peer-reviewed literature and EIS guidelines (external scan) and an environmental scan in the form of a survey with selected EIS (internal scan), followed by the selection of meaningful indicators for quality care in EIS.
2. Design and setup of a digital infrastructure for our RLHS to collect data routinely and iteratively regarding selected indicators of quality care in EIS.
3. Implementation of the RLHS data capture platform in real-life settings while systematically and continuously analyzing data to generate new evidence and recommendations for improvement of the RLHS.
4. Use of RLHS digital technologies to collect data, perform analysis, and propose recommendations for subsequent clinical care as well as capacity-building activities tailored to evolving needs in individual EIS as identified by the data collected.
5. Evaluation of outcomes related to clinical practice and program-level changes.
6. Evaluation of overall outcomes of the RLHS and dissemination of findings to key stakeholders.

### Study Settings

The RLHS literature recommends small-scale pilot-testing of digital technologies to build knowledge and confidence regarding complex digital systems as such innovations are often viewed skeptically by health care clinicians and managers [27,33,34]. For this reason, we purposefully selected a maximum variation sample of 11 EIS among the 33 existing EIS in Quebec based on various characteristics: environment (academic and nonacademic), setting (urban, semiurban, and rural), years of operation (<5 years vs >10 years), and patient age range covered by admission criteria (adolescents only, young adults only, or both; Table 1). EIS were also selected for their willingness to improve services and to represent diversity in relation to the previously identified implementation challenges they have faced [21]. All 11 EIS invited to the study agreed to participate, although 18% (2/11) mentioned staffing problems as a potential barrier to full participation in the project. These EIS were retained as staffing is an important issue in real-world implementation. As *early adopters*, these EIS will guide implementation and future scale-up of the RLHS. Representatives of the 11 selected EIS participated in activities leading to the development of this protocol and in project implementation activities.

**Table 1:** Characteristics of the selected sites.

EIS^a^ for psychosis	Location of the EIS: urban, semirural, or rural	Years of operation	Active service users, N	Full-time staff, N
1	Urban	>10	290	16
2	Urban	>10	220	10
3	Urban	>10	150	12
4	Urban	>10	45	2
5	Urban	>10	270	14
6	Urban	>10	180	3
7	Urban-semirural	<5	190	10
8	Semirural	>10	30	4
9	Semirural	<5	130	10
10	Semiurban and rural	>10	60	3
11	Urban and semirural	<5	130	7

^a^EIS: early intervention services.

### Objective 1: Assess Feasibility and Acceptability of an RLHS in EIS

#### Phase 1: Identifying Indicators for the RLHS for EIS Through an External and Internal Scan and Building the RLHS Community (Completed)

**Figure 3:**
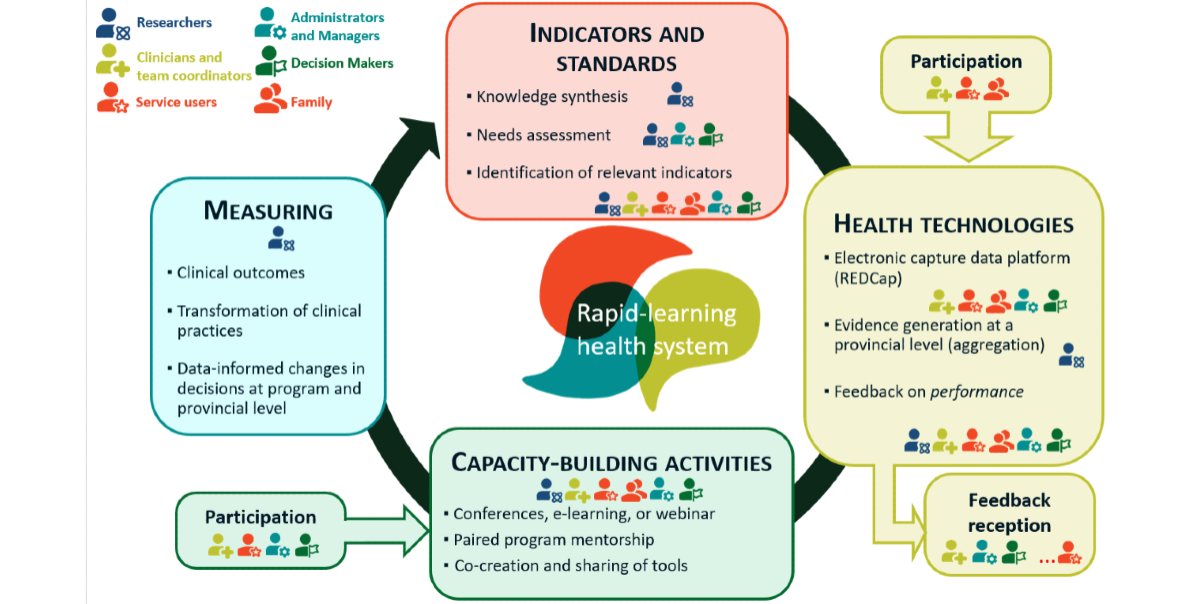
Involvement of stakeholders in our rapid-learning health system for early intervention for psychosis.

Quality indicators are measures or metrics based on guidelines or health organization directives used in monitoring the quality of patient care [40,41]. The research team identified indicators based on extensive literature reviews, including an external environmental scan of published national and international EIS guidelines and fidelity scales, and the peer-reviewed literature on program evaluation and outcomes in EIS [20,22,42]. The team then conducted an internal environmental scan using an email survey (unpublished data) inviting clinicians, team leaders, local decision makers and managers from participating EIS, and other key stakeholders (service users, caregivers, researchers, and representatives from the *Centre national d’excellence en santé mentale*, Quebec Ministry of Health and Social Services) to prioritize the indicators by importance, document the degree of implementation for each indicator in their respective EIS, estimate the capacity to improve implementation with the available resources, and determine the availability and level of data already collected for each indicator. We also assessed what resources would be needed in each EIS for measurement of the designated indicators. In total, 2 group discussions were convened by videoconference with representatives of the stakeholder groups representing the various EIS to gather input, as shown in Figure 3.

Table 2 provides the final list of evidence-based indicators and corresponding data collection procedures. In keeping with the RLHS requirements, we chose measurable indicators (eg, delay between referral and initial evaluation; a scale for self-rated clinical outcomes). These indicators were also chosen to balance maximum impact on program quality and patient outcomes with minimal burden related to data collection for the participating EIS.

**Table 2:** List of indicators and examples of data collected for each indicator.

Indicators	Examples of data collected
Service user engagement and satisfaction with services	Services adapted to the needs of young people Youth-friendly environment Disengagement Outreach practices Youth satisfaction
Family engagement	Type of intervention offered Percentage of families reached Number of visits Satisfaction of family members
Access to care—pathways	Direct access Referral sources, including self and the community Inclusion and exclusion criteria Number of contacts before access
Access to care—systemic delays	Time between referral and First contact First assessment Start of treatment
Continuous education	Number and type of continuing education events attended by workers Supervision and mentoring
Provider-to-patient ratios	Patient: Mental professional ratio Patient: Psychitriest ratio
Evidence-based practices and recovery-oriented practices	Cognitive behavioral therapy, family intervention, employment or study programs, integrated treatment for substance use disorders, and peer support Type of specialists who offer the interventions Percentage of patients receiving long acting injectable antipsychotics Percentage of patients receiving clozapine
Self-reported outcomes by the patient	Patient’s evaluation of their health, recovery, and quality of life

#### Phase 2: Designing the RLHS by Building Digital Infrastructure (Completed)

Program-level indicator data are collected using the REDCap (Research Electronic Data Capture; Vanderbilt University) digital platform, which provides an open-access, user-friendly, secure electronic health data capture platform for routinely collecting real-time clinical data. Hosted by the *Centre de Recherche du Centre Hospitalier de l’Université de Montréal*, REDCap allows the team leader of each participating EIS to collect program-level data. The platform is accessible from any electronic device (eg, computer, tablet, or smartphone) through a secure, open-access website. Each EIS can independently import data by answering specific multiple-choice and open-ended questions on selected indicators.

Data from service users and family members on the quality of services, an often-neglected indicator in the literature, are collected during on-site, web-based, or outreach clinical appointments. Each participating EIS collects information on service user satisfaction using the *Happy or Not* wireless digital terminals conveniently located on the walls of waiting rooms. Alternatively, service users can access the questionnaire on the web using any electronic device through a bar code scan. The questionnaire includes 3 questions. The first asks the following: *Are you satisfied with the service you received today?* Using 4 smiley-face emoticon buttons on the terminals, service users respond by choosing a face indicating whether they are *very happy*, *happy*, *unhappy*, or *very unhappy* with the service they received. The second question asks the following: *Among the following items, which one did you appreciate the most/least: quality of care and services, being welcomed with respect, feeling listened to, waiting time, respect for my opinion, something else?* These items were selected based on a literature review of youth-friendly mental health services and prioritized by consensus with service user representatives. Finally, comments are solicited using an open textbox.

A second REDCap-supported digital questionnaire for a more comprehensive evaluation of service quality and self-evaluation of personal recovery dimensions may be completed on the web or with any electronic device using a bar code scan or weblink. This quality-of-service digital questionnaire provides service users and family members with access to either the same questionnaire as the one on the service user feedback terminal with the 4 smiley-face emoticons (1-2–minute duration) or a more detailed version (10-minute duration). Satisfaction with the most recent on-site, web-based, or outreach clinical appointment may be evaluated, as well as service users’ perceptions dating from the beginning of the EIS. Finally, service users can rate their satisfaction with their health situation, quality of life and recovery, and the impact of services on their recovery journey.

#### Phase 3: Implementing the RLHS Data Capture Platforms (Completed) and Feedback Development (Ongoing)

New digital technologies (eg, the REDCap digital platform for EIS clinicians, smiley-face emoticon feedback terminals, bar code scans, and REDCap satisfaction with services questionnaire) were presented to key stakeholders for comments, and adjustments were made before deployment. The technologies were then tested with at least two representatives from each stakeholder group (service users, family members, EIS coordinators, and managers) to ensure clarity of content and effectiveness of the digital tools. Learning from these usability testing activities was compiled and used by the RLHS project coordinator during web-based or on-site meetings with EIS managers, coordinators, or leaders to support easy, safe, and effective uptake of the RLHS. This implementation strategy leads to high program engagement and strengthens partnerships between researchers, experts, clinical staff, managers, and EIS leaders.

#### Phase 4: Using RLHS Digital Technologies to Collect Data, Perform Analysis, and Share Results and Feedback With EIS and All Stakeholders (Ongoing)

##### Data Collection and Support

The RLHS collects data on selected indicators from each participating EIS at 4-month intervals using the REDCap platform (Table 2). Maintaining regular or as-needed contact with each EIS via web conferencing, telephone, email, or in person, the project coordinator supports participating EIS with data collection, use of the REDCap platform, and integration of the collected data into clinical routines. EIS leaders and coordinators enter data on organizational indicators (eg, number of clinical staff, caseload, and referral sources) and evidence-based interventions offered (eg, cognitive behavioral therapy, supported employment, and family interventions) directly into the REDCap digital platform.

##### Continuous, Real-Time Feedback to the EIS on Quality Indicators for Essential Components

After completion of the quarterly data collection cycle by the clinical team leader, the RLHS provides feedback to each EIS in the form of an individualized, user-friendly graphic report generated quarterly. Progress on specific program-level indicators can be tracked by each EIS over time, and its implementation level can be compared with the aggregated data from other participating EIS and with provincial standards. This feedback indicates whether the EIS meets or does not meet the provincial benchmarks for each specific indicator, providing the rationale for each essential component and guidance on how to improve implementation. The RLHS then uses the feedback system to guide subsequent actions toward better-informed and evidence-based implementation [28]. Moreover, the aggregated data on service user and family perceptions of quality and satisfaction with services, including their self-assessments of progress toward clinical recovery, are integrated into the REDCap digital platform, allowing the RLHS to provide regular service user feedback to the individual EIS.

The EIS may receive feedback reports on services and the service user or family satisfaction *happy or not* questionnaire by email or through a website, selecting a preferred frequency (eg, daily, weekly, or monthly). The EIS may also monitor their overall progress for selected periods (eg, daily, weekly, monthly, or quarterly). These 2 reports may be used for administrative reporting; advocacy work to secure resources; guidance; support for quality improvement in services; or descriptions of clinical services tailored to service users, families, or other audiences.

#### Phase 5: Evaluating Outcomes Related to Change at the Program Level Based on Capacity-Building Activities (Ongoing)

Capacity building is understood as an evidence-driven process for strengthening the abilities of individuals, organizations, and systems to perform core functions effectively, efficiently, and sustainably, continuously improving and developing them over time [43]. Capacity-building activities are geared toward helping program managers, clinical team leaders, and clinicians use data effectively to improve the quality of clinical practices, aligning them with guidelines and tailoring practices to data-identified program needs. These activities take the form of knowledge exchange events for improving knowledge and clinical skills while providing program representatives and stakeholders with opportunities to share their experiences, increase self-assessment skills, and participate more fully in the RLHS.

The AQPPEP and the *Centre national d’excellence en santé mentale* of the Quebec Ministry of Health and Social Services have been partners in designing this project. Project-related webinars and web-based training with participating EIS occur roughly 3 times a year and are conducted with program leaders, coordinators, or managers of each participating EIS. Clinical teams from participating EIS are met by the RLHS research team or representatives from the *Centre national d’excellence en santé mentale*, who explain the project, examine the EIS feedback reports in further detail highlighting strengths and challenges of the EIS, and discuss the rationale behind essential components and alternative ways of reaching goals. In addition to these meetings involving all participating EIS, we are partnering with the *Centre national d’excellence en santé mentale* to provide individualized digital training and coaching to improve EIS performance on specific indicators and developing a web-based media library for asynchronous training on related themes. Programs demonstrating high performance on certain indicators (positive deviance) may be partnered with programs needing help. The *Centre national d’excellence en santé mentale*, the Ministry of Health and Social Services, and AQPPEP already use this type of system for peer mentorship. These approaches have proven effective for use in knowledge translation and implementation science [43-45]. A continuous back-and-forth between digital data capture, continuous feedback on performance, and capacity-building activities will facilitate positive evolution in aligning participating Quebec EIS with best practices.

#### Phase 6: Evaluating and Disseminating RLHS Outcomes to Stakeholders (to Be Implemented)

The RLHS project and its outcomes will be presented at AQPPEP events, which are attended by most staff from the Quebec EIS, and at Quebec, Canadian, and international scientific conferences. We plan to adapt the RLHS based on lessons learned from this pilot project in terms of successes, weaknesses, facilitators, and challenges. The anticipated longer-term structural impact of the project will be the adoption and integration of the RLHS by EIS across the province, ideally with support from the Ministry of Health and Social Services. The project will positively affect decision-making at the local and provincial levels to become more data-informed and responsive in real time. Institutional bodies housing many of the EIS will be better able to monitor their implementation, targeting areas for improvement and resources needed. The Ministry of Health and Social Services will be able to follow the progress of EIS implementation across the province in relation to changes in sociopolitical measures and context (eg, investments, provision of new guidelines, or revisions to existing guidelines). The AQPPEP, the *Centre national d’excellence en santé mentale* of the Ministry of Health and Social Services, and similar organizations currently structured to train and support EIS will become more resource-efficient and effective after using the RLHS by tailoring their offerings to EIS, selecting appropriate target groups for training, and adopting data-driven evaluation and modification in capacity-building activities.

### Objective 2: Assess the Impact of the RLHS in EIS

The RLHS will further provide us with valuable information and data suggesting whether this paradigm does indeed lead to improved quality of care in EIS. The RE-AIM framework, used to assess the feasibility and impact of our project, was developed specifically to evaluate the implementation of interventions in real-world settings and sensitize researchers, knowledge users, and stakeholders to the essential elements involved in the sustainable adoption and implementation of targeted interventions. For our RLHS, we will assess *reach* (proportion of the targeted population that participates in the RLHS), *effectiveness* (impact of the RLHS on outcomes), *adoption* (extent and ease of adoption of the RLHS and degree of change), *implementation* (in-depth analyses of RLHS process data to determine which facilitators and barriers are associated with better implementation of the RLHS), and *maintenance* (extent to which the RLHS and its impact can be maintained), as shown in Figure 1.

To gain a qualitative perspective, we will invite all stakeholder groups (clinicians, managers, service users, and family members), advisory committee members, and representatives from the selected EIS (clinical staff, program leaders, managers, and decision makers) to participate in focus groups. Before the end of the project, a total of 6 remote focus groups (8-10 participants per group and 1.5-hour duration) will be implemented as follows: 1 (20%) for clinicians, 2 (40%) for program leaders (one for medical professionals and the other for professional team leaders), 1 (20%) for managers and decision makers, 1 (20%) for service users, and 1 (20%) for family members. The focus groups will be held by videoconference with a trained moderator and a research staff member acting as cofacilitators. Focus group questions will be designed following Krueger and Casey [46] and structured to explore the 5 key dimensions of learning health systems by Lessard [37] that capture the nature of an RLHS: the *goals* pursued by an RLHS to promote evidence-based and quality care; the *social dimension*, focused on building a community; the *technical dimension*, addressing digital data integration into routine care; the *scientific dimension*, enabling learning, innovation, and discovery; and the *ethics dimension*, ensuring that an RLHS pursues its learning and innovation activities in a manner that protects patient rights and privacy. Focus group participants will provide information on their experiences and perceptions related to the RLHS; the impact of the RLHS on them; their willingness to change and maintain use of the RLHS; attitudes about data collection; and the facilitators and barriers to implementation encountered, including their impact on decision-making at both the clinical and administrative levels. A research assistant will transcribe the focus group audio files and prepare them for analysis. Informed by the Braun and Clark analytic procedure [47], we will (1) familiarize ourselves with the data (reviewing transcriptions for accuracy), (2) generate initial codes using the dimensions of learning health systems by Lessard [37], (3) review and redefine themes, and (4) further unpack the analysis through the writing process.

For a quantitative and qualitative picture of EIS evolution along the RE-AIM parameters, we will track the uptake of the RLHS and extract data on all indicators from the REDCap platform, monitoring performance for each EIS on each indicator (Table 2) and comparing data from baseline to project completion to assess effectiveness. The components of the RE-AIM framework will be assessed as outlined in Textbox 2.

**Assessment guidelines for each component of the Reach, Effectiveness, Adoption, Implementation, and Maintenance framework.**

**Reach**

- Proportion of invited early intervention services (EIS) that participate in the project
- Proportion of invited EIS representatives (eg, clinicians, team leaders, or managers) and invited service users and family members who participate in capacity-building activities, knowledge exchange events, and implementation meetings
- Proportion of participating EIS who adopt our electronic data capture platform and ask service users and family members to provide information on satisfaction with services, self-evaluation of recovery dimensions, and the impact of services on recovery
- Proportion of invited people from each stakeholder group (clinicians, managers, service users, and family members) who participate in research focus groups

**Effectiveness**

- Improvement over time in indicators (eg, reduction of delays in access, increase in service user and family member engagement in services, satisfaction with services, and recovery outcomes such as employment)
- Increase over time in provision of evidence-based care as required by Ministry guidelines—the *cadre de référence* (eg, proportion of EIS offering cognitive behavioral therapy, family interventions, supported employment or education, integrated interventions for substance use disorder, peer support, and pharmacological interventions)
- Accuracy of data obtained from each EIS throughout the project using the rapid-learning health system (RLHS) electronic platform based on a comparison of the program-reported data from REDCap (Research Electronic Data Capture; Vanderbilt University) surveys in our RLHS with data collected by chart review on a selection of charts from each participating EIS. Deidentified data on *access to care* (eg, referral sources and delay from referral to initial evaluation), interventions offered (eg, cognitive behavioral therapy and family psychoeducation), and indicators of user engagement will be collected by research participants from the charts of 20 randomly selected service users at baseline and an additional 10 service users at all other time points (4 months preceding study onset and every 4 months subsequently until study completion). This step will ensure the trustworthiness of self-report data from the EIS by comparing self-report data with objective data from the files (eg, delays in evaluation and percentages of service users offered family interventions). If trustworthy, data reported by the programs themselves, as in our RLHS, may enable the creation of large, ecologically valid data sets that may be used to draw inferences about program performance and its relationship with patient outcomes on different recovery dimensions
- Perceptions of each stakeholder group (clinicians, managers, service users, and family members) regarding the ability of the RLHS to promote evidence-based and quality care in the EIS

**Adoption**

- Proportion of programs represented and proportion of each invited stakeholder group (clinicians, team leaders, managers, service users, and family members) in attendance at the various training sessions offered by the project
- Number of programs not involved in the research project whose representatives express interest in adopting the RLHS after attending presentations at the *Association québécoise des programmes pour premiers épisodes psychotiques* (Quebec Association of Programs for First-Episode Psychosis) or other events
- Progression over time in the proportion of data collected by program staff and service users as well as completion rates
- Proportion of participating EIS that continuously engage service users and family members to provide information on satisfaction with services, self-evaluation of recovery dimensions, and the impact of services on recovery using our electronic data capture platform
- Perceptions of each stakeholder group (researchers, clinicians, managers, service users, and family members) regarding the ability of the RLHS to foster a learning community
- Perceptions of each stakeholder group (clinicians, managers, service users, and family members) on whether it was feasible for the EIS to integrate indicators and digital data into routine care

**Implementation**

- Extent to which capacity-building strategies (eg, training) are implemented (at least one targeted after each 4-month data collection period)
- Proportion of participating programs using RLHS health technologies regularly until the end of the project
- Barriers, facilitators, and overall burden related to implementation of the RLHS as assessed qualitatively in focus groups
- Perceptions of each stakeholder group (clinicians, managers, service users, and family members) regarding the feasibility of implementing the RLHS in EIS
- Perceptions of each stakeholder group (clinicians, managers, service users, and family members) regarding the extent to which the RLHS protects patient rights and privacy

**Maintenance**

Maintenance is defined as the use of health technologies over time, with regular data collection by programs estimated in terms of the extent to which data collection is sustained by the participating programs over the course of the project

- Program commitment (e-survey) to continue using the electronic data capture system beyond the project
- Proportion of EIS attending advisory committee meetings over the entire duration of the project
- Proportion of EIS attending capacity-building and knowledge exchange events over the entire duration of the project
- Perceptions of each stakeholder group (clinicians, managers, service users, and family members) on how the RLHS enables learning, innovation, and discovery

### Ethics Approval

In August 2019, this proposal was accepted by the *Fonds de recherche du Québec-Santé*. Research ethics approval was received from the ethics board of the *Centre de Recherche du Centre Hospitalier de l’Université de Montréal* in December 2019 (reference 19-282 and MP-02-2020-8627), followed by institutional ethics approval from each of the 11 participating sites. Any important modifications to the protocol were reported to the ethics board of the *Centre Hospitalier de l’Université de Montréal* as well as the institutional research ethics boards overseeing the participating sites.

At all sites, youth, family members, and professionals provided web-based or written consent to participate in the study according to the protocol and to the regulations governing informed consent procedures.

## Results

Phase 1 was implemented between September 2018 and December 2018 to inform the project proposal, which was submitted to a Quebec government granting agency, the *Fonds de recherche du Québec-Santé*, in December 2018. On the basis of a previous descriptive study by our group characterizing all Quebec EIS [21], we selected 11 EIS representing the different contexts in which EIS services are delivered (Table 1). They all agreed to participate in the RLHS project. In phase 1, the authors first performed a knowledge synthesis of relevant peer-reviewed literature on essential EIS components, guidelines, and performance indicators. On the basis of this knowledge synthesis, 8 meaningful indicators for quality care in EIS (Table 2) were chosen through a survey and consensus meetings with representatives of each stakeholder group, including those from each of the 11 participating sites. An environmental scan in the form of a survey was then sent to the clinical leaders of the 11 selected sites to estimate capacity and assess their support needs regarding implementation of the RLHS, especially the capacity for data collection.

Phase 2 was implemented between September 2019 and May 2020. It involved the creation (with the collaboration of service users, team leaders, and researchers) and setup of the RLHS digital infrastructure using the REDCap platform and digital terminals that allowed service users and clinical team leaders to collect data routinely and iteratively on the selected indicators of quality care.

Phases 3 to 5 are ongoing and will continue for the first 6 months of 2022. The RLHS data capture platforms were first made available to the 11 EIS in June 2020, allowing for data collection on the selected indicators. These data are systematically and continuously analyzed to generate new evidence and recommendations for improving the RLHS as well as user-friendly illustrated feedback on a few indicators for the participating EIS and all stakeholder groups. The collected data also inform capacity-building activities tailored to the evolving needs of individual EIS and those of the 11 EIS as a group.

Phase 6, which assesses the impact of the RLHS (objective 2) and the dissemination of our findings, will be implemented in 2022. Using the RE-AIM framework, we will evaluate the outcomes related to clinical practice and program-level changes to assess the overall impact of the RLHS in EIS. In this regard, quantitative and qualitative data will be analyzed.

## Discussion

At the completion of the project, we should have developed the first province-wide database for real-time, clinically relevant data on quality indicators from representative EIS. We also expect that clinical practices at participating EIS will be better aligned with provincial and international EIS guidelines. Program capacity for continuous data collection and quality improvement in services and care provision will increase. Importantly, access to services by users and families and satisfaction with services should also improve, leading to better recovery outcomes for individual patients.

Should results of the RLHS project prove effective, we will have the potential to immediately scale up this RLHS across the province given the strong links between this project and Quebec EIS and the credibility of the project with the AQPPEP. We will also count on government support as a financial partner on the grant, including our ongoing support from the Quebec Ministry of Health and Social Services dating from the beginning of the grant submission process. Our decision to develop free, open-access instruments and platforms is another advantage. Further dissemination of the RLHS will result in population-level improvements in outcomes for psychosis. Over the longer term, should the type of RLHS we propose take root across the province, Quebec may rapidly advance to become both a national and international exemplar in EIS.

This project will also have multiple structural impacts. The first is an increase in the provision of patient-centered care, using individual-level data to tailor treatments while offering program-level data to improve patient and family experiences bearing on the accessibility, quality, and responsiveness of EIS. The second area of impact will affect the overall system of care across Quebec EIS, creating, most importantly, a system that continuously *learns*. The system as a whole and each individual EIS will have developed an increased capacity for providing evidence-based care, monitoring its own performance, setting improvement targets, using data to make program-level decisions, using aggregated data to make provincial-level decisions, and generating greater capacity for collaborative learning and multistakeholder interactions. By the end of this pilot project, the RLHS for EIS will be ready for deployment to all the remaining EIS in Quebec.

Finally, lessons from this project may support provincial decision-making regarding health informatics solutions, health care monitoring, system integration, the creation of communities of practice, and multicenter research. Most importantly, this project can contribute to a better understanding and operationalization of the RLHS approach in mental health and health services. Moreover, this project will lay the foundation for extending the RLHS paradigm to other Canadian provinces and to other countries where EIS for psychosis programs are currently available.

## Abbreviations

AQPPEP: Association québécoise des programmes pour premiers épisodes psychotiques (Quebec Association of Programs for First-Episode Psychosis)
EIS: early intervention services
RE-AIM: Reach, Effectiveness, Adoption, Implementation, and Maintenance model
RLHS: rapid-learning health system
